# The Provincial Baseline of PM_2.5_ in China and Its Hierarchical Management Strategy

**DOI:** 10.3389/fpubh.2022.908760

**Published:** 2022-07-12

**Authors:** Doudou Jin, Shaojie Kong, Changhong Ou, Anwei Chen, Fei Li

**Affiliations:** ^1^College of Resources and Environment, Hunan Agricultural University, Changsha, China; ^2^School of Information and Safety Engineering, Zhongnan University of Economics and Law, Wuhan, China

**Keywords:** cumulative frequency curve method, PM_2.5_ baseline, hierarchical management strategy, China, air quality

## Introduction

Atmospheric PM_2.5_ pollution has become a challenge worldwide, especially in some developing countries (1–3). PM_2.5_ has become the primary pollutant in most cities in China (4) and attacked more and more scholars' attention (5–8). In recent years, although the PM_2.5_ pollution in some areas of China shows a slight decrease, which does not alleviate this serious problem (9). If more accurate and effective control measures are taken, the PM_2.5_ concentration (population-weighted) will gradually reduce to 35 μg/m3 (China's current air quality standard) in 2030 (10). Although the Chinese government has taken many ways to deal with PM_2.5_ pollution, air quality is still not optimistic, especially in winter (4). The definitions of pollution in China are based on the air quality standard value (10 μg/m3) formulated by the World Health Organization (WHO) or national level I concentration limit (15 μg/m3) and national secondary concentration limit (35 μg/m3). However, the size of the human population and their activities, and the level of economic development in each region are quite different. Therefore, using the “One-Size Fits All” policy for PM_2.5_ control on a relatively large scale is obviously unscientific (11, 12).

The introduction of baseline value in PM_2.5_ control can effectively overcome the shortcomings mentioned above. The baseline is the upper limit of element content in a certain area under the influence of human activities (calculated under low human activities). There are similarities and differences between baseline, background, and standard values. The background value is the statistical average of the quality parameters obtained from monitoring relatively clean areas in a region. The standard value is formulated by relevant national laws and regulations to protect and improve the living environment and ecological environment to ensure human health. However, environmental standards cannot replace the baseline due to the different geological conditions, geochemical characteristics, and human activities in different regions. In baselines investigation, the methods in geochemical baseline investigation are widely referenced. However, the geochemical background and geochemical baseline are often confused or replaced by each other in concept and application in many situations. For many people, the differences between them are ignored. Cannon, head of the Yellowstone geochemical baseline research project in the United States, once pointed out that the geochemical background represents the concentration of elements in natural substances, excluding the impact of human activities (13). On the contrary, the baseline represents the concentration of elements measured in time in some places in areas disturbed by human activities, which is usually not the actual background. A geochemical baseline is a natural change in the concentration of chemical elements in the earth's surface materials. However, this natural change does not exclude the impact of human activities. It aims to describe the current supergene environmental conditions and is a benchmark material to measure future environmental changes. It has greater practical significance and can provide an essential basis for future environmental risk assessment. The normalization method and cumulative frequency curve method are the most commonly used methods for determining geochemical baseline (14). At first, many scientists used the normalization method to study the concentration of elements in marine and estuarine sediments. Later, it was introduced into the study of geochemical baseline, so its scope of application is limited. The cumulative frequency curve method has a long history. It can determine the baseline value of any substance. Therefore, this paper uses the cumulative frequency curve method to explore determining the Chinese baseline values of PM_2.5_ on a provincial scale.

The primary aims of this work are to establish the baseline values of PM_2.5_ in 31 provinces across China using the cumulative frequency curve method. Then, the provinces are classified according to the relationship between baseline values, monitoring values, and standard values. Finally, the PM_2.5_ hierarchical management strategy is proposed for the three types of provinces obtained.

## Methods of the Cumulative Frequency Curve

Bauer et al. devised the cumulative frequency curve approach to assess geochemical baselines (15, 16). The method involves plotting the distribution curve on the decimal axis with the element concentration as the X-axis and the cumulative frequency as the Y-axis. A curve usually has one or two inflection points (points where the curve has a significant deflection). Below the inflection point represents the lower limit of human activities, and the baseline value was obtained by averaging the data less than the inflection point. If the cumulative frequency curve was nearly straight without an inflection point, the average of all data was utilized as the baseline value (17). Certainly, when the cumulative frequency curve method is used in establishing the PM_2.5_ baseline, some uncertainties or differences need to be explained. Firstly, according to the relevant research, the calculation of element geochemical baseline value using this method requires long time-series data. While, due to Spatio-temporal pollution characteristics of PM_2.5_, the daily data for 2018 and 2019 is preliminarily selected and the effect of time series length can be further discussed. Secondly, this work selected the provincial scale, so we should later consider whether the application details of this method will change if the scale changes under different environmental control decision needs.

## Data Sources

PM_2.5_ data are obtained from China National Environmental Monitoring Center (http://www.cnemc.cn/). Due to the unavailability of data from Hong Kong, Macao, and Taiwan, data from 31 provinces on the mainland were analyzed.

## PM_2.5_ Determination of Baseline Value

**Figure 1:**
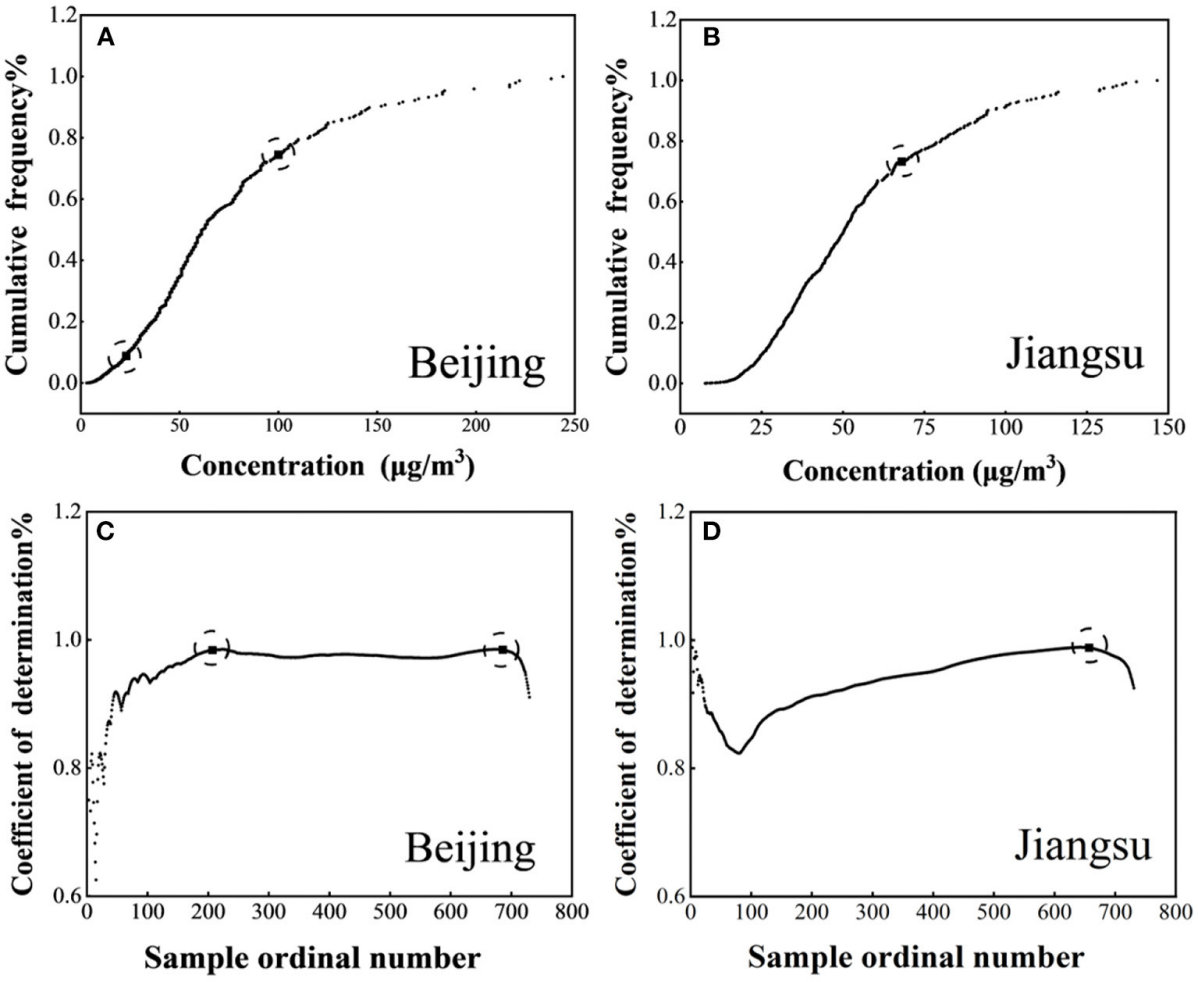
Cumulative frequency distribution **(A,B)** and inflection point **(C,D)** of Beijing and Jiangsu.

The first step is to draw the cumulative frequency curve. The second step is gradually calculating the fitting curve's determination coefficient between each point and the first point. Then, a chart was obtained using the ordinal number as the x-axis and the determination coefficient as the y-axis. First, the determination coefficient will fluctuate and then reach the relatively high point (higher than the data surroundings), the first inflection point of the cumulative frequency curve. Subsequently, the determination coefficient may reach the highest point and the second inflection point again. Take Beijing and Jiangsu for examples (Figures 1C,D). Beijing's coefficient of determination began with a series of fluctuations and then gradually rose to the highest point, the first inflection point of Beijing's cumulative frequency curve. Starting from the first inflection point, the second inflection point can be obtained in the same way. The coefficient of determination of Jiangsu also has a series of fluctuations at first and then increases to a maximum point, and then there is no increase in fluctuation. Therefore, the cumulative frequency curve of Jiangsu has only one inflection point. The cumulative frequency curves of Beijing and Jiangsu are shown in Figures 1A,B.

After determining the inflection point, the next step is to determine which inflection point is used as the upper limit of the baseline value. When the cumulative frequency curve has only one inflection point, the average value of all points below the inflection point shall be taken as the baseline value. When the cumulative frequency curve has two inflection points, compare the similarity between the frequency distribution between the two inflection points and the frequency distribution before and after the first inflection point. If it is close to the first inflection point, select the first inflection point as the upper limit of the calculated baseline value. Otherwise, select the second inflection point.

It can be seen from Table 1 that the cumulative frequency curve of 93.55% of provinces (29 provinces) has two inflection points, indicating that human activities have a significant impact on PM_2.5_ emission. It can be known from the provincial population data (which could be found from the data availability link), that the population of these provinces in 2018 and 2019 is also relatively large. The annual average concentrations limit of China air quality standard (GB3095-2012) and WHO phase I target (IT1) are 35 μg/m^3^. Comparing the baseline value, monitoring value, and the standard value of each province one by one, it is found that the average value of 2-year monitoring value of 61.29% of provinces and the baseline value of 29.03% of provinces exceed this value. All baseline values are lower than the average value of the 2-year monitoring value. Based on this, it is very unscientific to adopt the same standard requirements for different provinces. In addition, since the baseline value is obtained under the condition of low human activities, it is tough to directly require PM_2.5_ emissions to be reduced to the same standard value for some provinces where the baseline value exceeds the standard value. For example, the baseline value of Anhui Province (39.44 μg/m^3^) is greater than the standard value. Therefore, it is being challenged to directly require it to reduce PM_2.5_ emissions to the standard value. Anhui Province can implement “step-by-step” measures to reduce the emission of PM_2.5_ to the baseline value first and then to the standard value.

**Table 1:** PM_2.5_ in each province baseline and two-year mean (μg/m^3^).

**Province**	**Inflection**	**Baseline**	**Average**	**Difference**	**Province**	**Inflection**	**Baseline**	**Average**	**Difference**
	**point**	**value**	**annual value**			**point**	**value**	**annual value**	
Guangxi	2	26.70	33.52	6.82	Anhui	2	39.48	47.64	8.16
Zhejiang	2	30.23	33.04	2.81	Beijing	2	37.38	46.12	8.74
Gansu	2	28.78	32.01	3.23	Jiangsu	1	36.41	43.48	7.07
Jilin	2	23.22	31.67	8.45	Shandong	2	36.01	45.91	9.9
Guangdong	2	27.40	28.95	1.55	Shaanxi	1	34.94	47.79	12.85
Inner Mongolia	2	23.31	27.24	3.93	Liaoning	2	34.42	39.45	5.03
Guizhou	2	23.56	26.95	3.39	Hubei	2	34.41	45.12	10.71
Qinghai	2	22.87	26.07	3.2	Hunan	2	32.92	40.65	7.73
Fujian	2	23.37	24.78	1.41	Jiangxi	2	32.17	36.12	3.95
Yunnan	2	19.22	22.67	3.45	Xinjiang	2	46.77	50.51	3.74
Hainan	2	13.07	15.86	2.79	Ningxia	2	30.94	34.65	3.71
Tibet	2	11.74	13.32	1.58	Chongqing	2	31	38.31	7.31
Henan	2	42.64	60.68	18.04	Sichuan	2	30.04	36.09	6.05
Tianjin	2	41.17	51.5	10.33	Shanghai	2	27.61	34.42	6.81
Hebei	2	40.78	52.91	12.13	Heilongjiang	2	21.68	37.97	16.29
Shanxi	2	39.77	50.64	10.87					

*1-one inflection point, 2-two inflection points; the difference between the baseline value and the annual mean value*.

## Classification Control Based on Three Values

**Figure 2:**
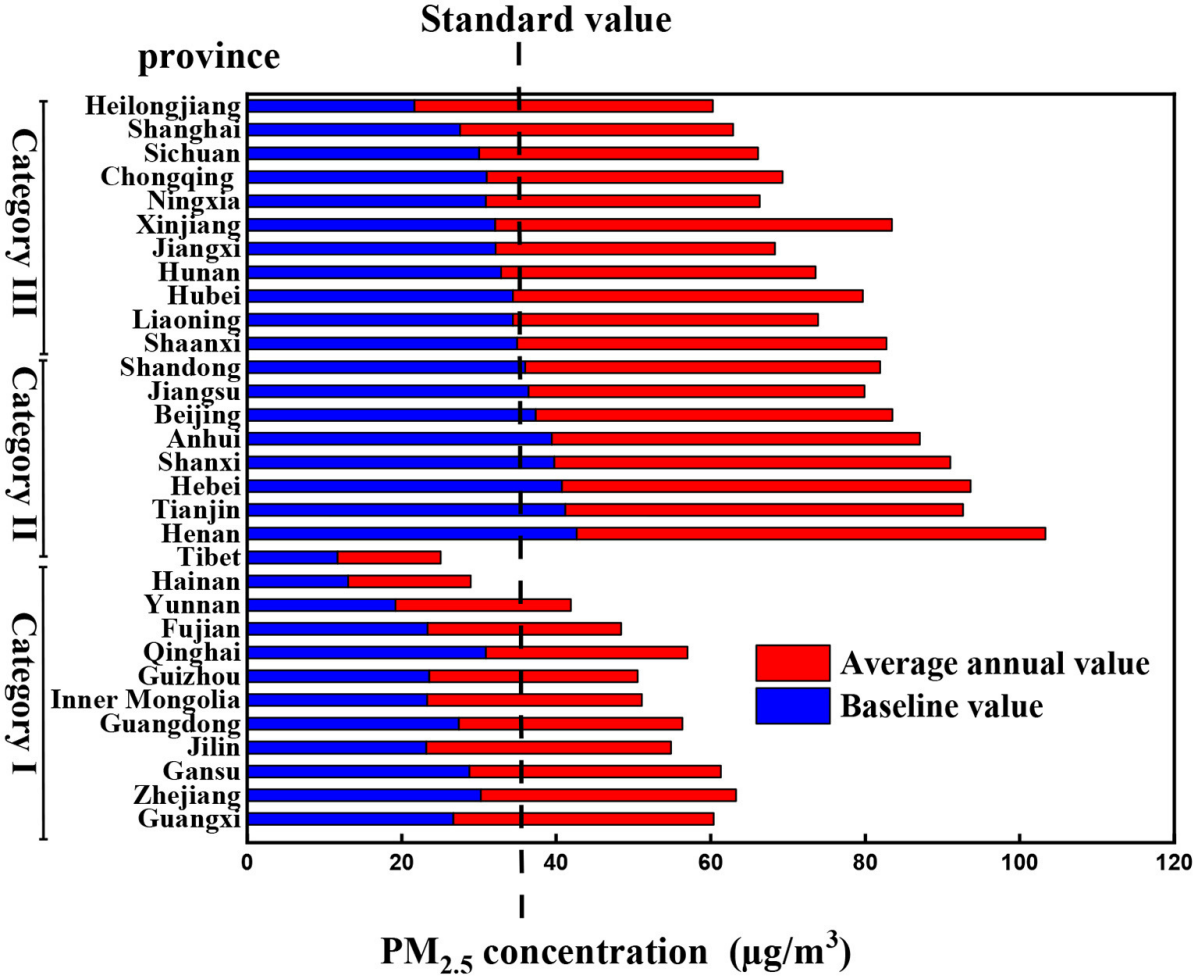
Classification diagram based on the relationship between baseline value, monitoring value, and standard value in 31 provinces.

According to the relationship between baseline, monitoring, and standard values, 31 provinces are divided into three categories for classified control (Figure 2). As shown in Figure 2, Guangxi, Zhejiang, Gansu, Jilin, Guangdong, Inner Mongolia, Guizhou, Qinghai, Fujian, Yunnan, Hainan, and Tibet belong to the first category. Henan, Tianjin, Hebei, Shanxi, Anhui, Beijing, Jiangsu, and Shandong belong to the second category; Shaanxi, Liaoning, Hubei, Hunan, Jiangxi, Xinjiang, Ningxia, Chongqing, Sichuan, Shanghai, and Heilongjiang belong to the third category. The first category's monitoring value and baseline value are all lower than the standard value. Among them, the baseline value of Zhejiang, Guangdong, Fujian, and Tibet is not much different from the average value of the 2 years. Therefore, while these provinces continue to maintain this state, they can further move closer to the baseline value and strive to minimize the emission of PM_2.5_. The baseline value and monitoring value of the second category are all higher than the standard value. Combined with the above, it can be seen that the baseline value is calculated under low human activities. If these provinces are directly required to reduce the emission of PM_2.5_ to the standard value, it is complicated and unscientific. Therefore, provinces belonging to this category can implement “step-by-step” measures, that is, reduce to the baseline value first and then reduce to the standard value. For provinces belonging to the third category, it is necessary to reduce the emission of PM_2.5_ to the national standard first and then strive to promote the green and high-quality development of the economy further to reduce the emission of PM_2.5_ to the baseline value.

## Conclusion

The baseline value of PM_2.5_ is determined by the cumulative frequency curve method. According to the relationship between baseline value, monitoring value, and national standard value (35 μg/m^3^), the PM_2.5_ hierarchical management strategy is proposed. Ninety-three percent of the provinces' PM_2.5_ emissions are significantly affected by different degrees of human activities. The provinces are divided into three categories, and different compliance requirements are put forward. The monitoring value and baseline value of the first category of provinces are all lower than the standard value. While these provinces continue to maintain this state, they can further move closer to the baseline value and strive to minimize the emission of PM_2.5_. The baseline value and monitoring value of the second category of provinces are higher than the standard value. Provinces in this category can implement “step-by-step” measures that are first reduced to the baseline value and then reduced to the standard value. The baseline value for the third category of provinces is lower than the standard value, while the monitoring value is higher. These provinces can first reduce the emission of PM_2.5_ to the national standard and then strive to promote the development of a green and high-quality economy to reduce the emission of PM_2.5_ further to reach the baseline value.

## Data Availability Statement

The original contributions presented in the study are publicly available. This data can be found here: https://doi.org/10.6084/m9.figshare.19779688.v1.

## Author Contributions

DJ and SK structured and wrote the manuscript. CO and SK analyzed the data and contributed with descriptive analysis. FL and AC reviewed the manuscript. All authors contributed to manuscript revision, read, and approved the submitted version.

## Funding

This study was funded by the National Natural Science Foundation of China (51879105), Hubei Provincial Outstanding Young Science and Technology Innovation Team Project (T2021032), and Fundamental Research Funds for the Central Universities from Zhongnan University of Economics and Law (2722021AJ007, 202151414, 202151408).

## Conflict of Interest

The authors declare that the research was conducted in the absence of any commercial or financial relationships that could be construed as a potential conflict of interest.

## Publisher's Note

All claims expressed in this article are solely those of the authors and do not necessarily represent those of their affiliated organizations, or those of the publisher, the editors and the reviewers. Any product that may be evaluated in this article, or claim that may be made by its manufacturer, is not guaranteed or endorsed by the publisher.
